# Efficacy of concurrent chemoradiotherapy alone for loco-regionally advanced nasopharyngeal carcinoma: long-term follow-up analysis

**DOI:** 10.1186/s13014-023-02247-y

**Published:** 2023-04-05

**Authors:** An-An Xu, Jing-Jing Miao, Lin Wang, An-Chuan Li, Fei Han, Xun-Fan Shao, Zhi-Wen Mo, Shao-Min Huang, Ya-Wei Yuan, Xiao-Wu Deng, Chong Zhao

**Affiliations:** 1 Department of Radiation Oncology, Affiliated Cancer Hospital and Institute of Guangzhou Medical University, No. 78, Hengzhigang Road, Yuexiu District, Guangzhou, 510095 Guangdong People’s Republic of China; 2 Department of Nasopharyngeal Carcinoma, Sun Yat-Sen University Cancer Center, State Key Laboratory of Oncology in South China, Collaborative Innovation Center for Cancer Medicine, Guangdong Key Laboratory of Nasopharyngeal Carcinoma Diagnosis and Therapy, 651 Dong Feng Road East, Guangzhou, 510060 China; 3 Department of Radiation Oncology, Fujian Medical University Union Hospital, Fuzhou, China; 4 Department of Radiation Oncology, Sun Yat-Sen University Cancer Center, State Key Laboratory of Oncology in South China, Collaborative Innovation Center for Cancer Medicine, Guangdong Key Laboratory of Nasopharyngeal Carcinoma Diagnosis and Therapy, 651 Dong Feng Road East, Guangzhou, 510060 China

**Keywords:** Nasopharyngeal carcinoma, Concurrent chemotherapy, Intensity-modulated radiotherapy, Prognostication, Toxicity

## Abstract

**Background:**

To analysis the clinical outcomes of concurrent chemoradiotherapy (CCRT) alone based on 10-year results for loco-regionally advanced nasopharyngeal carcinoma (LANPC), so as to provide evidence for individualized treatment strategy and designing appropriate clinical trial for different risk LANPC patients.

**Methods:**

Consecutive patients with stage III-IVa (AJCC/UICC 8th) were enrolled in this study. All patients received radical intensity-modulated radiotherapy (IMRT) and concurrent cisplatin chemotherapy (CDDP). The hazard ratios (HRs) of death risk in patients with T3N0 was used as baseline, relative HRs were calculated by a Cox proportional hazard model to classify different death risk patients. Survival curves for the time-to-event endpoints were analyzed by the Kaplan–Meier method and compared using the log-rank test. All statistical tests were conducted at a two-sided level of significance of 0.05.

**Results:**

A total of 456 eligible patients were included. With 12-year median follow-up, 10-year overall survival (OS) was 76%. 10-year loco-regionally failure-free survival (LR-FFS), distant failure-free survival (D-FFS) and failure-free survival (FFS) were 72%, 73% and 70%, respectively. Based on the relative hazard ratios (HRs) of death risk, LANPC patients were classified into 3 subgroups, low-risk group (T1-2N2 and T3N0-1) contained 244 patients with HR < 2; medium-risk group (T3N2 and T4N0-1) contained 140 patients with HR of 2 – 5; high-risk group (T4N2 and T1-4N3) contained 72 patients with HR > 5. The 10-year OS for patients in low-, medium-, and high-risk group were 86%, 71% and 52%, respectively. Significantly differences of OS rates were found between each of the two groups (low-risk group *vs.* medium-risk group, *P* < 0.001; low-risk group *vs.* high-risk group, *P* < 0.001; and medium-risk group *vs.* high-risk group, *P* = 0.002, respectively). Grade 3–4 late toxicities included deafness/otitis (9%), xerostomia (4%), temporal lobe injury (5%), cranial neuropathy (4%), peripheral neuropathy (2%), soft tissue damage (2%) and trismus (1%).

**Conclusions:**

Our classification criteria demonstrated that significant heterogeneity in death risk among TN substages for LANPC patients. IMRT plus CDDP alone maybe suitable for low-risk LANPC (T1-2N2 or T3N0-1), but not for medium- and high-risk patients. These prognostic groupings provide a practicable anatomic foundation to guide individualized treatment and select optimal targeting in the future clinical trials.

**Supplementary Information:**

The online version contains supplementary material available at 10.1186/s13014-023-02247-y.

## Background

There were 133,354 new cases of nasopharyngeal carcinoma (NPC) worldwide in 2020, accounting for 0.7% of all cancers [1]. But its geographical global distribution is extremely unbalanced; over 70% of new cases are in east and southeast Asia, with an age-standardized rate (world) of 3.0 per 100,000 in China [2]. Unfortunately, over 75% of patients present with LANPC at the time of diagnosis [3]. Cisplatin-based CCRT has been established as the foundation of treatment strategy for LANPC based on several prospective randomized clinical trials and meta-analysis [4–7]. Recently, phase III randomized controlled trials have proved that induction chemotherapy (IC) or adjuvant chemotherapy (AC) added to CCRT can significantly prolong survival [4, 8–14]. However, not all patients of LANPC benefit from IC or AC [15, 16]. In addition, regimens with more chemotherapy has been demonstrated to associate with increasing risk of therapeutic toxicity [17]. Therefore, it is necessary to analysis the treatment strategies in LANPC with different failure risks, so as to facilitate individualized treatment and avoid excessive toxicity causing by overtreatment or treatment failure due to undertreatment.

Because LANPC contains a heterogeneous group of patients, which led to broadly varying disease extent. It is suggested that the current anatomy-based staging system is insufficient for prediction prognosis or treatment benefits. Studies have assessed whether incorporating other clinical factors and non-anatomical factors, such as gross tumor volume [18], 18F-fluorodeoxyglucose (18F-FDG)- positron emission tomography (PET) standardized uptake value (SUV) within primary tumor [19, 20] and plasma Epstein-Barr virus DNA (EBV-DNA) load [21, 22], etc*.* in LANPC patients would present various disease characteristics, and lead to different outcomes when used a uniform treatment. However, because these parameters had established in different hospitals and laboratories, their cut-off values are not standardized, which limits their clinical application as a general indicator for screening tumor heterogeneity.

AJCC/UICC TNM staging classification is an internationally recognized staging system that is widely used for predicting prognosis, guiding treatment strategy for different risk groups and facilitating exchange of experience between oncology centers. But significant heterogeneity is often observed for different T-N subgroups within equivalent clinical TNM stage [23, 24]. It is reasonable to select pertinent chemotherapy scheme for different failure risk levels. Therefore, in order to enhance the power to detect a survival benefit with additional IC or AC, studies excluded those LANPC patients with low relapse risk, some excluded patients with T3-4N0M0 [14, 25], and the other excluded T3-4N0/T3N1M0 [26]. But it is still questioned whether the current division is the optimal scheme, especially within the updated 8th stage groupings. On the basis of this premise, we conducted this retrospective analysis of a series of LANPC patients treated with CCRT. We constructed a framework according to the risk of death after treatment, intended to provide high sensitivity to predict overall survival (OS) risk using the 8th edition T and N stage, so as to provide better guidance of individualized treatment and to serve as a skeleton framework for additional biomarkers be employed.

## Methods

### Patient eligibility

A total of 456 pathologically diagnosed LANPC patients who received CCRT alone between August 2001 and June 2014 in one attending group of Sun Yat-sen University Cancer Center were included in this study. The pretreatment workup included a complete history and physical examination, hematological and biochemical profiles, nasopharyngoscopy, chest X-ray, abdominal ultrasound, magnetic resonance imaging (MRI) of the head and neck, and whole body emission computed tomography (ECT). Positron emission tomography computed tomography (PET/CT) with 18F-FDG was conducted for 80 (17.5%) patients. Patients were excluded if they had stage I–II disease, distant metastasis (DM) disease, missing medical data, not finish 2-cycle CCRT, received IC or AC. And all patients were restaged according to the AJCC/UICC stage classification system, 8th edition [27].

### Treatment

All eligible patients received curative IMRT. The details of the whole IMRT process have been described previously [28]. Briefly, gross tumor volume was determined according to diagnostic MRI as well as physical examination. The nasopharynx gross tumor volume (GTVnx) and the gross tumor volume of metastatic neck lymph nodes (GTVnd) were identified. Two clinical target volumes (CTVs) were delineated: CTV1 and CTV2. CTV1 was defined as the GTVnx plus a 5 to 10 mm margin (2 to 3 mm margin posteriorly) to encompass the high-risk sites of microscopic extension and the whole nasopharynx. CTV2 was defined as the CTV1 plus a 5 to 10 mm margin (2 to 3 mm margin posteriorly) to encompass the low-risk sites of microscopic extension, the level of the lymph node located, and the elective neck area. The prescription dose was 68–70 Gy, 60–66 Gy, 60 Gy, and 54 Gy, in 30 fractions, for the planning target volumes (PTVs) derived from GTVnx, GTVnd, CTV1, and CTV2, respectively. PTVs were generated by a geometric circumferential expansion of 3 mm, as per our institutional protocol that is determined by the aggregation of systematic and random errors. The dose constrains to organs at risk (OARs) were within the tolerance according to the QUANTEC [29]. The dose limitation to OARs has been detailed described in our previous published study [30].

All patients received 2 cycles cisplatin concurrent chemotherapy. The regimen consisted of cisplatin alone at 80 mg/m^2^/d, intravenous infusion on days 1 and 22 over 2 h. The cumulative cisplatin dose (CCD) was 160 mg/m^2^.

### Patient assessment and follow-up

Patients were evaluated at least once per week during IMRT. The first assessment of tumor response was performed one month after completion of radiotherapy by physical examination and flexible nasopharyngoscopy. MRI of the head and neck was performed three months after radiotherapy. Then, patients were required to be evaluated once every 3 months in the first 3 years, once every 6 months for the following 2 years, and once every year thereafter. During each follow-up visit, complete physical exams including indirect nasopharyngeal speculum examinations were performed. Head-neck MRI, hematologic and biochemical profiles, chest radiography and abdominal ultrasonography were required each year. Further investigations would be arranged when clinically indicate. Tumor recurrence or metastasis was confirmed on the basis of the results of biopsy or fine-needle aspiration. For lesions that was not accessible, the clinical diagnosis was based on the presence of at least 2 radiological features on CT, MRI, ECT or ^18^F-FDG PET/CT. Management of residual disease and tumor relapse, if detected, was determined on a case-by-case basis.

Acute toxicities during CCRT were graded according to the Common Terminology Criteria for Adverse Events (version 3.0); late toxicities were graded according to the Late Radiation Morbidity Scoring Criteria of the Radiation Therapy Oncology Group [31].

### Statistical analysis

We calculated failure-free survival (FFS) from the date of first histological diagnosis to the date of treatment failure or death from any cause, whichever was first. Histology biopsy or at least two different examinations (MRI, CT, PET/CT or endoscopy) were used to determine treatment failure. Overall survival (OS) was defined as death due to any cause. For locoregional (LR-FFS) analysis, we recorded the latencies to the first locoregional recurrence, or death from any cause. For distant failure-free survival (D-FFS), we recorded the latencies to the first distant failure, or death from any cause. Statistical analysis was performed using SPSS 22.0 (Chicago, Illinois, USA). Survivals were calculated by the Kaplan–Meier method. The log-rank test was used to calculate the significance of differences between survival curves. Cox regression was used to determine prognostic factors. Hazard ratios (HRs) and 95% confidence intervals (CIs) were calculated by a Cox proportional hazards model. All statistical tests were conducted at a two-sided level of significance of 0.05.

## Results

### Patient characteristics

From August 2001 and June 2014, 456 patients were enrolled, with the detailed demographic and clinical characteristics summarized in Table 1. The majority were nonkeratinizing (undifferentiated) carcinoma (438, 96.1%). After being restaged using the AJCC/UICC 8th staging system, 302 patients were stage III and 154 were stage IVa. Up to then, 298 (65%) survivors were followed up to at least 10 years.

**Table 1:** Characteristic of 456 nasopharyngeal carcinoma patients

Characteristics	No. of patients (N = 456)	%
Age		
Median	46	
Range	11–75	
Sex		
Male	344	75.4
Female	112	24.6
Karnofsky scale		
≥ 80%	456	100
< 80%	0	0
Histology (WHO)		
II	18	3.9
III	438	96.1
UICC/AJCC 8th		
T-classification		
T1	7	1.5
T2	29	6.4
T3	284	62.3
T4	136	29.8
N-classification		
N0	83	18.2
N1	213	46.7
N2	140	30.7
N3	20	4.4
Clinical stage		
III	302	66.2
IVa	154	33.8

All patients completed IMRT as planned. The median D95 of PTVnx, PTVnd, PTV1, and PTV2 were 69.2 Gy, 63.6 Gy, 64.8 Gy, and 56.1 Gy, as shown in Additional file 1: Table S1.

### Survivals

At the last follow-up on May 30th, 2021, the median follow-up was 12-year (IQR 6 years—14 years). Overall, One hundred and thirteen patients died, among them, 84 (74.3%) were cancer-specific deaths, and 29 (25.7%) patients died of noncancer-related causes. The 5-year and 10-year OS was 81% and 76%, respectively. 137 (30%) of 456 patients had treatment failure. The proportion of patients with FFS at 5-year and 10-year were 74% and 70%, respectively. 35 (25.5%) of 137 patients experienced local recurrence, 8 (5.8%) had regional recurrence, and 72 (52.6%) developed DM. The 10-year LR-FFS and D-FFS were 72% and 73%, respectively.

### Relative HRs according to various T and N combination subgroups for death

**Fig. 1:**
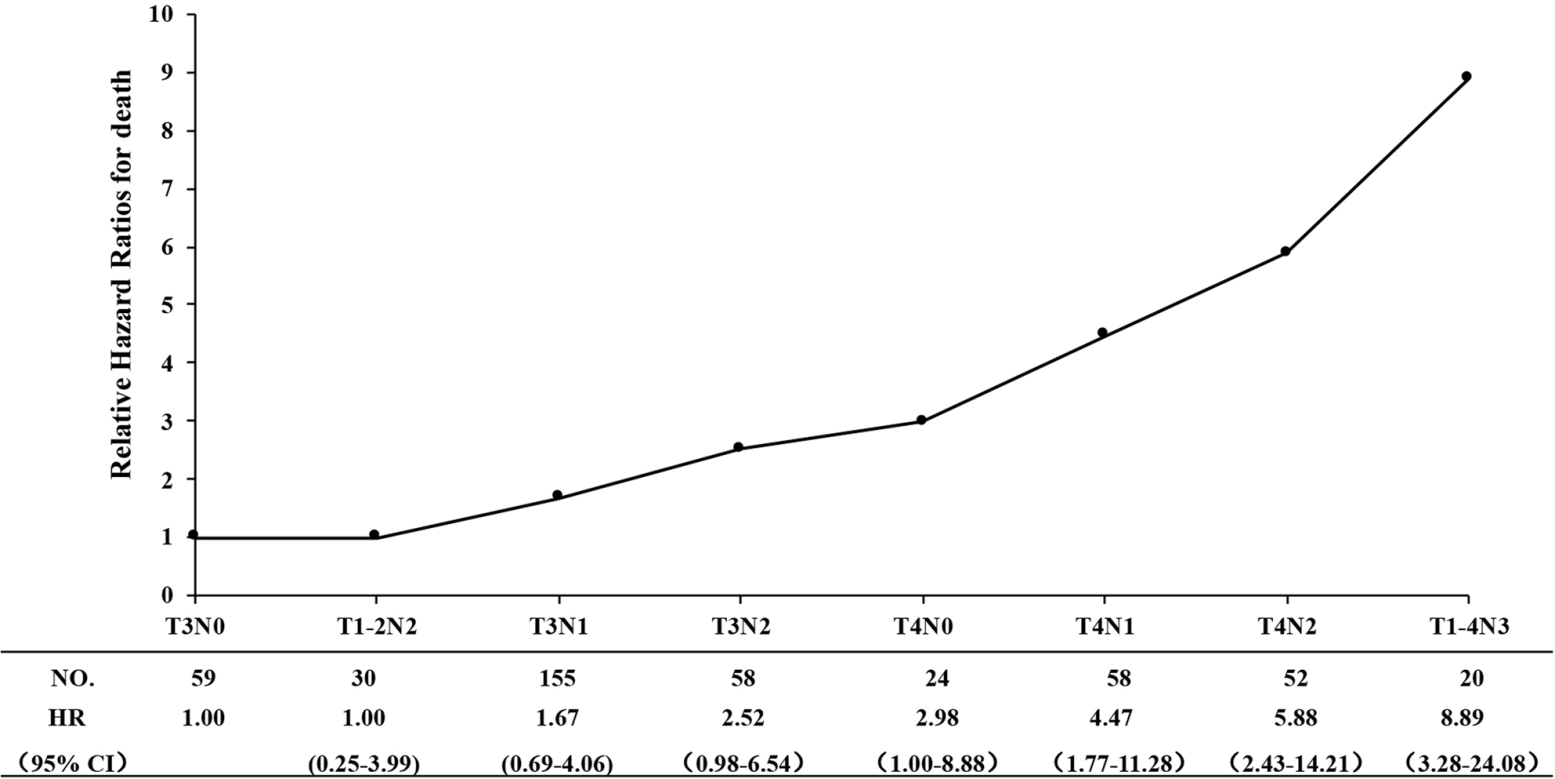
Relative Hazard ratios (HRs) of different T and N combination subgroups for overall death

To evaluate the relative risk of death for different T and N subgroups, the HRs for each subgroup were calculated with death risk as the endpoint. T1N2 was analyzed with T2N2 because of the limited case fold. Similarly, any T with N3 were combined and analyzed. The HR of patients with T3N0 disease was defined at baseline (HR = 1). Host factors (sex, age) were included as covariates, and the Cox regression model was used to calculate HRs in various T and N subgroups. Patients were then divided into 8 subgroups. As T and N combination increased, the HR showed a tendency to gradually increase (Fig. 1).

### Comparison on treatment outcomes among risk subgroups for LANPC

**Fig. 2:**
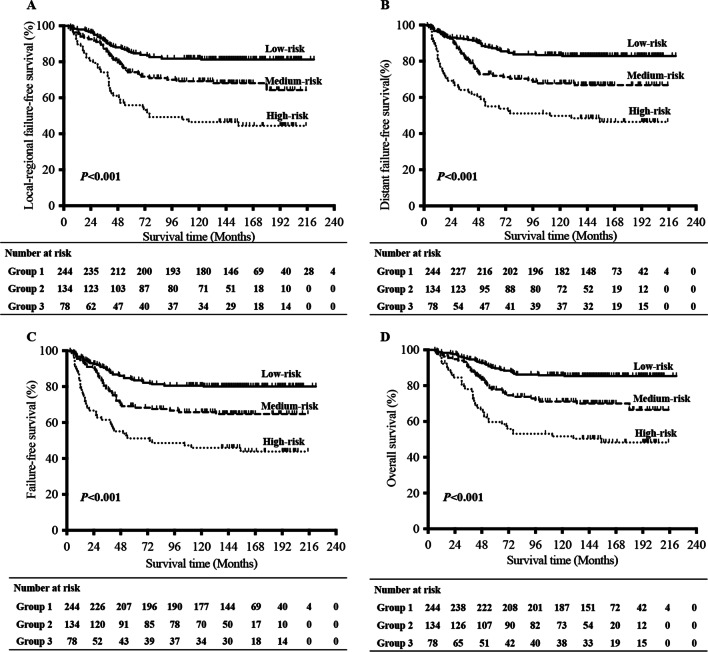
Survival curves in different sub-groups. **A** Loco-regionally failure-free survival. **B** Distant failure-free survival. **C** Failure-free survival. **D** Overall survival. *P* values were calculated with the unadjusted log-rank test

According to the HR analysis, 456 patients were classified into 3 sub-groups: low-risk group (T1-2N2 or T3N0-1) contained 244 patients with HR < 2, medium-risk group (T3N2 or T4N0-1) included 134 patients with HR of 2–5, high-risk group (T4N2 or T1-4N3) involved 78 patients with HR > 5. The 10-year LR-FFS for low-risk, medium-risk and high-risk group were 82%, 69%, and 47%, respectively (*P* < 0.001). The 10-year D-FFS for low-risk, medium-risk and high-risk group were 83%, 68%, and 50%, respectively (*P* < 0.001). The 10-year OS for low-risk, medium-risk and high-risk group were 86%, 71%, and 52%, respectively (*P* < 0.001) (Fig. 2). Further analysis showed that when the OS rate was compared between each of the two groups (low-risk *vs.* medium-risk, low-risk *vs.* high-risk, and medium-risk *vs.* high-risk), significant differences could be found (*P* < 0.001, *P* < 0.001, and *P* = 0.002, respectively) (Table 2).

**Table 2:** Comparisons of survival outcomes in subgroups of LANPC patients

Characteristic	3-year survival (%)	5-year survival (%)	10-year survival (%)	Group 1		Group 2	
				χ^2^	*P* value	χ^2^	*P* value
**LR-FFS**	87	77	72				
Group 1	92	85	82				
Group 2	87	74	69	9.08	**0.003**		
Group 3	74	56	47	45.01	** < 0.001**	10.71	**0.001**
**D-FFS**	84	78	73				
Group 1	92	87	83				
Group 2	82	73	68	12.86	** < 0.001**		
Group 3	64	55	50	47.91	** < 0.001**	9.99	**0.002**
**FFS**	82	74	70				
Group 1	90	84	81				
Group 2	79	69	66	10.90	**0.001**		
Group 3	62	51	46	45.94	** < 0.001**	10.61	**0.001**
**OS**	91	81	76				
Group 1	95	90	86				
Group 2	90	78	71	13.49	** < 0.001**		
Group 3	78	60	52	50.56	** < 0.001**	9.39	**0.002**

*LR-FFS* local–regional failure-free survival, *D-FFS* distant failure-free survival, *FFS* failure-free survival, *OS* overall survival

*P* < 0.05 was considered statistically significant.

Group1: T1-2N2 and T3N0-1, Group 2: T3N2 and T4N0-1, Group 3: T4N2 and T1-4N3

### Failure patterns in risk subgroups

The different failure patterns in risk subgroups were summarized in Table 3. The results showed a direct relationship between an increased distant metastasis rate or death rate and a higher risk subgroup (*P* < 0.001 and *P* < 0.001, respectively). More than half of the high-risk patients (52%) died.

**Table 3:** Failure patterns in subgroups

Failure site	Low-risk (n = 244)			Medium-risk (n = 134)			High-risk (n = 78)			*P*
	No. of patients	%		No. of patients	%		No. of patients	%		
Locoregional recurrence	21	9		8	6		12	15		0.066
Distant metastasis	24	10		26	19		22	28		** < 0.001**
Loco-regional and distant metastasis	5	2		0	0		3	4		0.106
Death	35	14		39	29		39	50		** < 0.001**

*P* < 0.05 was considered statistically significant

### Adverse events

Over the entire treatment course, 174 patients (38%) experienced grade 3 or 4 acute adverse events. Leukopenia was the most common severe hematologic event (21%), following by neutropenia (14%). Mucositis was the most common severe nonhematologic event (17%). The incidence of late toxic effects of grade 3 or 4 was 16% (72 patients). There were 40 patients (9%) suffered more than one late toxic effects of grade 3 or 4. Ear damage (8%) was the most common severe late toxic event. The detailed acute and late toxicities are shown in Table 4.

**Table 4:** Grade 3–4 adverse events

Acute adverse events			Late adverse events		
Events	Grade 3	Grade 4	Events	Grade 3	Grade 4
Hematologic			Temporal lobe injury	25 (5%)	0
Anaemia	9 (2%)	0	Cranial neuropathy	18 (4%)	0
Thrombocytopaenia	0	0	Peripheral neuropathy	9 (2%)	0
Neutropenia	62 (14%)	5 (1%)	Eye damage	0	0
Leukopenia	98 (21%)	1 (< 1%)	Ear (deafness/otitis)	35 (8%)	4 (1%)
Nonhematologic			Bone necrosis	0	0
Dermatitis	16 (4%)	0	soft tissue damage	11 (2%)	0
Mucositis	78 (17%)	7 (2%)	Trismus	6 (1%)	0
Dysphagia	19 (4%)	0	Dry mouth	16 (4%)	0
Nausea	23 (5%)	0			
Vomiting	40 (8%)	0			
Dry mouth	9 (2%)	0			

## Discussion

As the first study with over 10-year follow-up using CCRT alone for LANPC in the era of IMRT, we established an anatomic-based risk stratification for overall death in LANPC patients, and generated three distinctly different risk groups: low-risk group (T1-2N2 and T3N0-1), medium-risk group (T3N2 and T4N0-1), and high-risk group (T4N2 and T1-4N3). Our results showed that low-risk patients can gain benefit from CCRT, with mild late toxicity; however, the 10-year OS rate was 71% for the middle-risk group, 52% for the high-risk group, which suggested that more advanced treatment strategy were needed for these patients.

Since the current TNM staging system included a heterogeneous group of NPC patients with different prognosis, important non-anatomical prognostic factors, such as EBV-DNA load or primary tumor SUV, are suggested to incorporate into guiding the clinical treatment [19–22]. However, the variation of cut-off values hamper their clinical applications. For example, in a matched study, the authors defined patients of N2-3 stage with an EBV DNA ≥ 4000 copies/ml as being very high-risk group [32]. Yet the results from a phase III study proved that the EBV DNA level ≥ 6000 copies/ml were significantly associated with poorer FFS and OS[14].

For low-risk group, our study showed a satisfactory 5-year OS (90%). A recent randomized trial selected low-risk LANPC patients treated with different CCD to test the non-inferiority of 2-cycle over 3-cycle concurrent cisplatin regimen. It concluded that 2-cycle concurrent cisplatin yielded comparable survival benefits to 3-cycle, and was associated with less acute and late toxicities and improved quality of life [33], which give us a clue that the satisfactory survival benefits might still be achieved by reducing CCD in concurrent chemotherapy for low-risk LANPC.

Over the past few decades, considerable efforts have been made to improve survival outcomes of LANPC patients by adding IC or AC to CCRT. Several phase 3 studies have shown that the additional IC or AC gained significant improvement in survivals compared with CCRT alone [4, 8–14]. In our study, the 5-year FFS and OS for medium-risk group was 69% and 78%, which were similar to that (66.4% and 77.7%) of CCRT alone group in Li et al. study[14]. Further, Li and colleagues proved that after adding IC to CCRT, a significant improvement of 5-year FFS and OS (77.4% and 85.6%, respectively) (*P* = 0.042) were noted [14], despite the complete rates of IMRT and ≥ 200 mg/m^2^ CDDP were lower than CCRT alone group (97.9% *vs.* 100%, and 85.9% *vs.* 98.3%, respectively). Therefore, for the medium-risk group, further investigation may be needed to evaluate the utility of IC or AC.

In the current study, the 10-year OS for the high-risk group (52%) were much lower than the low-risk group (86%) and medium-risk group (71%) (*P* < 0.001, *P* = 0.002, respectively). In addition, A series of phase 3 studies indicated that the IC + CCRT cannot effectively improve the prognosis of high-risk LANPC patients [13, 14, 34], further intensification treatment is needed. Recently, two phase 3 trials have focusing on the efficacy and safety profile of additional metronomic adjuvant capecitabine in patients with high-risk LANPC [26, 35]. Both trials proved that adjuvant capecitabine was well tolerance. Miao and colleagues [35] reported an estimated 7.7% (88.8% for adjuvant capecitabine *vs* 81.1% for CCRT) better 3-year DMFS and an estimated 11.5% (91.5% for adjuvant capecitabine *vs* 80% for CCRT) better 3-year LRRFS with the addition of capecitabine. Similarly, Chen and colleagues [26] reported an estimated 9.6% better 3-year FFS with the addition of AC (85.3% for metronomic capecitabine *vs* 75.7% for IC + CCRT), suggesting a potential role for capecitabine as an adjuvant therapy in the treatment of high-risk LANPC.

Anti-programmed death (PD) therapy has become the backbone of cancer immunotherapy and a major modality of cancer treatment. Since studies have proved that anti-PD1 therapy is a potential treatment option for patients with recurrent or metastatic (R/M) NPC [36–39]. It is reasonable to assume that it might also workable in LANPC. Several randomized trials are currently underway to evaluated therapeutic benefits of adding PD-1 antibody in high-risk LANPC, and the results are worth expected.

In this study, we summarized patients’ acute and late toxicities, especially the latter. Most of patients’ late toxicities were in grade 1–2, only 8% patients had grade 3–4 deafness or otitis, 5% patients had grade 3 temporal lobe injury, 4% patients had grade 3 dry mouth, 4% patients had grade 3 cranial neuropathy. Other grade 3–4 late toxicities were lower than 3%.

Besides, there are some limitations in this study. First, the data were derived from one single institution in endemic area, whether the findings can be reproduced and are generalizable to other patient populations remains to be demonstrated. External validation in multicenter hospitals is warranted. Second, some may argue that we did not integrate non-anatomical factors, such as EBV-DNA, into the model. However, routine detection of plasma EBV-DNA was not widely used for our patients treated before 2009, and the methodology has not been standardized so far. Our model presents a practical way for evaluating risk of death, and provides a relatively stable anatomic framework that partitions according to a death risk hierarchy.

## Conclusions

This article proved that our classification criteria are practicable and useful for LANPC. Cisplatin-based CCRT satisfied survival outcomes for low-risk LANPC patients. For medium-risk and high-risk patients, more effective systematic treatment strategies and treatment sequences need to be explored.

## Supplementary Information

**Additional file 1**. Volume and dosimetry data of target volumes.

## Data Availability

Not applicable.

## Abbreviations

NPC: Nasopharyngeal carcinoma
HRs: Relative hazard ratios
RT: Radiation therapy
IMRT: Intensity-modulated radiotherapy
2D-CRT: 2-Dimensional conventional radiotherapy
CCRT: Concurrent chemo-radiotherapy
AC: Adjuvant chemotherapy
IC: Induction chemotherapy
LR-FFS: Locoregional failure-free survival
D-FFS: Distant failure-free survival
DMFS: Distant metastasis-free survival
FFS: Failure-free survival
DFS: Disease-free survival
OS: Overall survival
DM: Distant metastasis
CT: Contrast-enhanced computed tomography
MRI: Magnetic resonance imaging
ECT: Emission computed tomography
PET: Positron emission tomography
SUV: Standardized uptake value
EBV-DNA: Epstein-Barr virus DNA
GTVnx: The gross tumor volume of nasopharynx
GTVnd: The gross tumor volume of the involved neck area
CTV1: The high-risk sites of microscopic extension
CTV2: The low-risk sites of microscopic extension
PTVs: Planning target volumes
OARs: Organs at risk
CCD: Cumulative cisplatin dose
CIs: Confidence intervals
