# Supplementary material for: Efficacy of concurrent chemoradiotherapy alone for loco-regionally advanced nasopharyngeal carcinoma: long-term follow-up analysis
Source: Radiat Oncol. 2023 Apr 5;18:63. doi: 10.1186/s13014-023-02247-y (PMC10074656; doi:10.1186/s13014-023-02247-y)
Supplement: Supplementary file 1 — Additional file 1. Volume and dosimetry data of target volumes. [file 13014_2023_2247_MOESM1_ESM.docx]

**Table S1. Volume and dosimetry data of target volumes**

|  | **Dmin, Gy** | **Dmax, Gy** | **Dmean, Gy** | **D95, Gy** | **D100, Gy** | **V100, %** |
| --- | --- | --- | --- | --- | --- | --- |
| **PTVnx** | 66.9 | 78.5 | 73.8 | 69.2 | 66.2 | 97.2 |
| **PTVnd** | 61.2 | 72.2 | 67.4 | 63.6 | 61.5 | 99.1 |
| **PTV1** | 59.1 | 78.5 | 68.2 | 64.8 | 58.8 | 99.0 |
| **PTV2** | 41.9 | 78.5 | 61.1 | 56.1 | 47.9 | 99.2 |
